# Validation of Messenger Ribonucleic Acid Markers Differentiating Among Human Acute Respiratory Distress Syndrome Subgroups in an Ovine Model of Acute Respiratory Distress Syndrome Phenotypes

**DOI:** 10.3389/fmed.2022.961336

**Published:** 2022-07-05

**Authors:** Karin Wildi, Kieran Hyslop, Jonathan Millar, Samantha Livingstone, Margaret R. Passmore, Mahé Bouquet, Emily Wilson, Gianluigi LiBassi, John F. Fraser, Jacky Y. Suen

**Affiliations:** ^1^Critical Care Research Group, Brisbane, QLD, Australia; ^2^Faculty of Medicine, The University of Queensland, Brisbane, QLD, Australia; ^3^Cardiovascular Research Group, Basel, Switzerland; ^4^Roslin Institute, University of Edinburgh, Edinburgh, United Kingdom

**Keywords:** acute respiratory distress syndrome, phenotypes, mRNA expression, up-and downregulation, precision medicine, predictive and prognostic enrichment, biomarker

## Abstract

**Background:**

The discovery of biological subphenotypes in acute respiratory distress syndrome (ARDS) might offer a new approach to ARDS in general and possibly targeted treatment, but little is known about the underlying biology yet. To validate our recently described ovine ARDS phenotypes model, we compared a subset of messenger ribonucleic acid (mRNA) markers in leukocytes as reported before to display differential expression between human ARDS subphenotypes to the expression in lung tissue in our ovine ARDS phenotypes model (phenotype 1 (Ph1): hypoinflammatory; phenotype 2 (Ph2): hyperinflammatory).

**Methods:**

We studied 23 anesthetized sheep on mechanical ventilation with observation times between 6 and 24 h. They were randomly allocated to the two phenotypes (*n* = 14 to Ph1 and *n* = 9 to Ph2). At study end, lung tissue was harvested and preserved in RNAlater. After tissue homogenization in TRIzol, total RNA was extracted and custom capture and reporter probes designed by NanoString Technologies were used to measure the expression of 14 genes of interest and the 6 housekeeping genes on a nCounter SPRINT profiler.

**Results:**

Among the 14 mRNA markers, in all animals over all time points, 13 markers showed the same trend in ovine Ph2/Ph1 as previously reported in the MARS cohort: matrix metalloproteinase 8, olfactomedin 4, resistin, G protein-coupled receptor 84, lipocalin 2, ankyrin repeat domain 22, CD177 molecule, and transcobalamin 1 expression was higher in Ph2 and membrane metalloendopeptidase, adhesion G protein-coupled receptor E3, transforming growth factor beta induced, histidine ammonia-lyase, and sulfatase 2 expression was higher in Ph1. These expression patterns could be found when different sources of mRNA – such as blood leukocytes and lung tissue – were compared.

**Conclusion:**

In human and ovine ARDS subgroups, similar activated pathways might be involved (e.g., oxidative phosphorylation, NF-κB pathway) that result in specific phenotypes.

## Introduction

More than five decades after its first description (1), acute respiratory distress syndrome (ARDS) still has an unacceptably high morbidity and mortality (2). After years of little success in ARDS research focusing on improving patient outcomes (3), analysis of large ARDS cohorts from randomized controlled trials identified two distinct biological subphenotypes amongst the heterogenous population of ARDS patients (4, 5): a discovery with potential for future targeted treatment in ARDS. In brief, a hypoinflammatory (or uninflamed; named P1) and a hyperinflammatory (or reactive; named P2) subphenotype, defined by specific functional and biological parameters, have been proposed and corroborated with existing retrospective clinical data (4–6). P2 was clearly associated with a more severe shock state and metabolic acidosis as well as worse clinical outcomes (4, 7). Importantly, the response of patients to specific medical measures and pharmacological treatment appears to be highly dependent on the respective subphenotype (4, 5, 8). Even though the concept of subphenotypes in ARDS is now widely accepted, there are still major gaps in our current knowledge regarding the in-depth comprehension of underlying biological driving factors and mechanisms.

Animal models have always played an important role in biological discovery and therapeutic translation in ARDS (9). We reported previously about ovine ARDS phenotype models that mimics some key features of human ARDS subphenotypes (10): phenotype 1 (Ph1: hypoinflammatory) and phenotype 2 (Ph2: hyperinflammatory). Additionally, a recent analysis revealed matching gene expression among animal models induced by lipopolysaccharides (LPS) as in our Ph2 model and the human P2 subphenotype (11).

To validate our ovine model on a molecular level, we compared a subset of messenger ribonucleic acid (mRNA) markers previously characterized in leukocytes from P2/P1 subphenotypes within the Dutch MARS (Molecular Diagnosis and Risk Stratification of Sepsis) cohort (12) with our ovine ARDS phenotypes model.

## Materials and Methods

### Animal Model

This study is a secondary analysis of pooled control animals of ovine Ph1 and Ph2 ARDS from two blinded, randomized, controlled preclinical trials. A more detailed explanation of the two studies is provided in the Supplementary Method.

Animal ethics was approved by The Queensland University of Technology Office of Research Ethics and Integrity (No 1600001108 and No 1800000606). All experiments were performed in accordance with the Australian Code of Practice for the Care and Use of Animals for Scientific Purposes and the Animal care and Protection Act 2001 (QLD).

**FIGURE 1:**
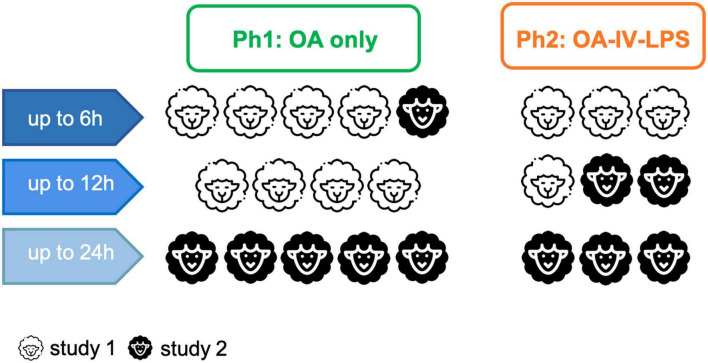
Overview of participating animals, observation time and their assignment to the respective study. Abbreviations: OA, oleic acid; OA-IV-LPS, oleic acid and lipopolysaccharide intravenously; Ph1, hypoinflammatory phenotype; and Ph2, hyperinflammatory phenotype.

A total of 23 female non-pregnant Merino-Dorset crossbreed ewes, aged 1–3 years, mean weight 49 kg (47–52), participated in this analysis and were randomly allocated to one of the two groups: Ph1 (*n* = 14) and Ph2 (*n* = 9); (10). Eight animals were observed up to 6 h (Ph1 *n* = 5, Ph2 *n* = 3), 7 animals up to 12 h (Ph1 *n* = 4, Ph2 *n* = 3) and 8 animals up to 24 h (Ph1 *n* = 5, Ph1 *n* = 3; Figure 1).

In short, after induction of general anesthesia and intubation, the animal was mechanically ventilated in a lung-protective fashion (13). After completion of instrumentation, the animal was turned in prone position and ARDS was induced: (1) animals randomized to Ph1 received oleic acid (OA; O1008; Sigma-Aldrich, Australia) sequentially in 0.03 ml/kg doses intravenously (IV) until a PaO_2_/FiO_2_ ratio (PF) of <150 mm Hg was reached (2) animals assigned to Ph2 received aforementioned oleic acid IV followed by 0.5 μg/kg of lipopolysaccharide (LPS: E. coli O55:B5, Sigma-Aldrich, Australia) infused over 1 h. Intra-experimental monitoring, management and data collection has been reported in detail previously (10, 14).

### Sample Collection and Processing

At study end, lung tissue was harvested and samples from the upper, middle and lower lobe of the right lung were stored in RNAlater (Invitrogen, United States) at 4^°^C overnight and then at –80^°^C until extraction.

For histopathological assessment, lung tissue was preserved in in 10% neutral buffered formalin for 24 h for embeddment in paraffin afterward, then tissue was sectioned to 5 μm thickness and stained with hematoxylin & eosin. Slides were assessed by an independent and blinded veterinary pathologist using the lung injury score (LIS) as recommended by the ATS for experimental ARDS in animal models (15). In short, the LIS scores neutrophils in the alveolar space (A), neutrophils in the interstitial space (B), hyaline membranes (C), proteinaceous debris filling the airspace (D), and alveolar septal thickening (E). Every item is given a score between 0 and 2. The LIS is then calculated by: [(20 × A) + (14 × B) + (7 × C) + (7 × D) + (2 × E)]/number of fields × 100, leading to a score between zero (no injury) and one (severe lung injury). Twenty random high-power fields (400× total magnification) were scored per section, and the LIS was calculated per animal (mean ± SD).

### Selected Messenger Ribonucleic Acid Markers

The 16 most upregulated mRNA markers in blood leukocytes in P2 and P1 subphenotypes were chosen as reported previously by Bos et al. (12). These were mainly markers of oxidative phosphorylation, cholesterol metabolism, and the Nuclear Factor Kappa-light-chain-enhancer of Activated B Cells pathway (NF-κB) as well as markers related to neutrophil and macrophage function in P2: matrix metalloproteinase 8 (MMP8), olfactomedin 4 (OLFM4), resistin (RETN), G protein-coupled receptor 84 (GPR84), CEA cell adhesion molecule 1 (CEACAM1), lipocalin 2 (LCN2), zinc finger DHHC-type palmitoyltransferase 18 (ZDHHC18), ankyrin repeat domain 22 (ANKRD22), CD177 molecule (CD177), and transcobalamin 1 (TCN1). For P1, mRNA markers involved in regulation of vital cell functions like proliferation and differentiation, motility and survival were among the most upregulated ones: membrane metalloendopeptidase (MME), retinol binding protein 7 (RBP7), adhesion G protein-coupled receptor E3 (ADGRE3), transforming growth factor beta induced (TGFBI), histidine ammonia-lyase (HAL), and sulfatase 2 (SULF2) were selected.

### Extraction of Messenger Ribonucleic Acid and Quality Control

Tissue from the upper, middle and lower regions of the right lung were independently homogenized in Trizol (Ambion, United States) using a Polytron PT 2100 (Kinematica AG, Switzerland). Following homogenization, a PureLink™ RNA Mini Kit (Invitrogen, United States) was used to purify total RNA and a DNase treatment was performed using a DNA-free™ Kit (Invitrogen, United States). Sample concentration and purity was determined using a NanoDrop 1000 Spectrophotometer (Thermo Scientific, United States) and RNA integrity number (RIN) was assessed on an Agilent Bioanalyzer 2100 (Agilent Technologies, United States; Supplementary Table 1). Samples with a RIN ≥ 6 were standardized to a concentration of 100 ng/μL in nuclease-free water. Final concentration was determined on a Qubit 2.0 Flurometer (Invitrogen, United States), before regional samples were pooled.

### Nanostring Gene Expression Analysis

A custom NanoString nCounter assay (NanoString Technologies, United States) was used to assess the expression of the 16 genes of interest and the six housekeeping genes (MAU2, POLR2A, PGK1, RPL19, TBP, and YWHAZ) used for data normalization. The assay consisted of a nCounter Elements TagSets (reporter and capture tags) and oligonucleotide probe pairs (reporter/probe A and capture/probe B) designed by NanoString Technologies. Oligonucleotides were supplied by Integrated DNA Technologies (Iowa, United States) as pools (one containing every Probe A and the other every probe B) at a final concentration of 5 nM per oligo and 25 nM per oligo, respectively. Probe pools were used to prepare the working pools described in subsequent steps, as per the manufacturer’s instructions. Prior to assay, pooled RNA samples were diluted in nuclease-free water to 20 ng/μL and concentration was measured using a Qubit 2.0 Flurometer (Invitrogen, United States). 140 ng of pooled RNA was combined with a master mix containing, nCounter Elements TagSets and oligonucleotide probe working pools (Integrated DNA Technologies, United States) in 0.2 mL PCR tubes, as per the manufacturer’s instructions (Supplementary Table 2). Samples were hybridized with reporter and capture probes at 67^°^C in a T100™ thermal cycler (Bio-Rad Laboratories, United States) for 18 h. To minimize potential evaporation, the thermal cycler lid was maintained 5^°^C higher than the block for the duration of the hybriziation. Following hybridization, 15 μL of RNAse-free water was added to the mixture. A total of 30 μL was loaded onto each lane of the NanoString microfluidic SPRINT™ cartridge (Seattle, United States) for automated processing on a nCounter SPRINT™ profiler (NanoString Technologies, United States).

### Data Analysis

The raw count data quality control assessment was conducted in nSolver™ Analysis Software 4.0 (NanoString Technologies, United States). Assay background correction was performed by subtracting the mean of the negative controls plus two standard deviations. Corrected counts were normalized to the geometric mean of the top 3 housekeeping genes (POLR2A, RPL19 and TBP), as determined by the geNorm algorithm. Normalization was performed using the nCounter Advanced Analysis 2.0 plugin.

Normalized mRNA counts are log2 transformed and levels were compared using a Mann–Whitney *U* test. Data in figures is expressed as mean and standard deviation (SD). All hypothesis testing was two-tailed and a *p*-value of less than 0.05 was considered statistically significant. All statistical analysis was performed using SPSS 27.0 (SPSS Inc, Chicago, United States).

## Results

### Studied Population

Baseline characteristics of experimental animals did not differ among Ph1 and Ph2 and are provided in Supplementary Table 3.

Clinical parameters during study observation period are presented at 6, 12, and 24 h of all animals alive at this time point (Supplementary Table 4). Ph2 animals displayed a higher heart rate and need for vasoactive drugs, indicating a more altered hemodynamic situation. While the PF ratio recovered to a certain degree in both injury models, Ph2 showed higher lactate levels combined with a more negative base excess, as well as lower platelet and higher neutrophil counts, pointing toward more disturbances in metabolic situation and more inflammation activation.

Levels of pro-inflammatory (IL-6 and -8) and anti-inflammatory cytokines (IL-10) were higher in Ph2 than Ph1 throughout the study observation time after induction of ARDS (Supplementary Figure 1).

The LIS was 0.28 (±0.06) in Ph1 and 0.35 (±0.06) in Ph2 (*p* 0.06 for comparison; Supplementary Figure 2). In addition, there were more alveolar thrombi present and more lung tissue was affected by necrosis in Ph2 animals.

### Levels of Messenger Ribonucleic Acid Markers Among Ovine Acute Respiratory Distress Syndrome Phenotypes All Time Points Pooled

**FIGURE 2:**
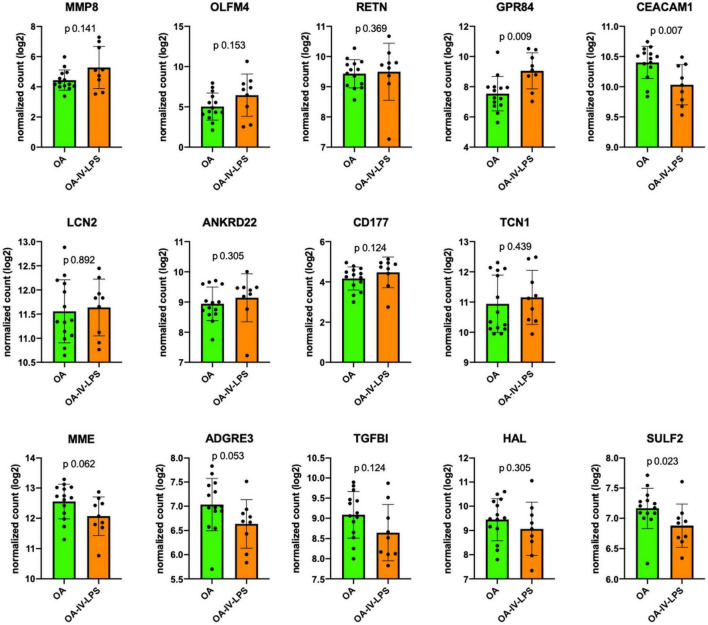
Comparison of mRNA expression markers between animals injured with OA (=Ph1) and OA-IV-LPS (=Ph2; in all animals, all time points of tissue harvesting). Figure legend: *y*-axis displays log2 normalized count of the respective mRNA marker at all sampling times (6 h: Ph1 *n* = 5, Ph2 *n* = 3; 12 h: Ph1 *n* = 4, Ph2 *n* = 3; 24 h: Ph1 *n* = 5, Ph2 *n* = 3). Error bar displays standard deviation, *p* value for comparison using Mann–Whitney *U* test. Abbreviations: OA, oleic acid; OA-IV-LPS, oleic acid and lipopolysaccharide intravenously; MMP8, matrix metalloproteinase 8; OLFM4, olfactomedin; RETN, resistin; GPR84G, protein-coupled receptor 84; CEACAM1, CEA cell adhesion molecule 1; LCN2, lipocalin 2; ANKRD22, ankyrin repeat domain 22; CD177, CD177 molecule; TCN1, transcobalamin 1; MME, membrane metalloendopeptidase; ADGRE3, adhesion G protein-coupled receptor E3; TGFBI, transforming growth factor beta induced; HAL, histidine ammonia-lyase; and SULF2, sulfatase 2.

Zinc finger DHHC-type palmitoyltransferase 18 and RBP7 were excluded because the counts were below the detection limit of the assay. Among the remaining 14 mRNA markers, in all animals over all time points, 13 markers showed similar trends in the ovine phenotypes as previously reported (12) in the MARS cohort (Figure 2): MMP8 (*p* 0.14), OLFM4 (*p* 0.15), RETN (*p* 0.37), GPR84 (*p* 0.009), LCN2 (*p* 0.89), ANKRD22 (*p* 0.31), CD177 (*p* 0.12), TNC1 (*p* 0.44) were more upregulated in Ph2 whereas MME (*p* 0.06), ADGRE3 (*p* 0.05), TGFBI (*p* 0.12), HAL (*p* 0.31), and SULF2 (*p* 0.02) expression was higher in Ph1. Only one mRNA marker, CEACAM1 (*p* 0.007), reported as upregulated in P2 in the MARS cohort, was consistently lower in Ph2 animals than in Ph1 at all time points.

### Levels of Messenger Ribonucleic Acid Markers According to Time Point of Tissue Harvesting

**FIGURE 3:**
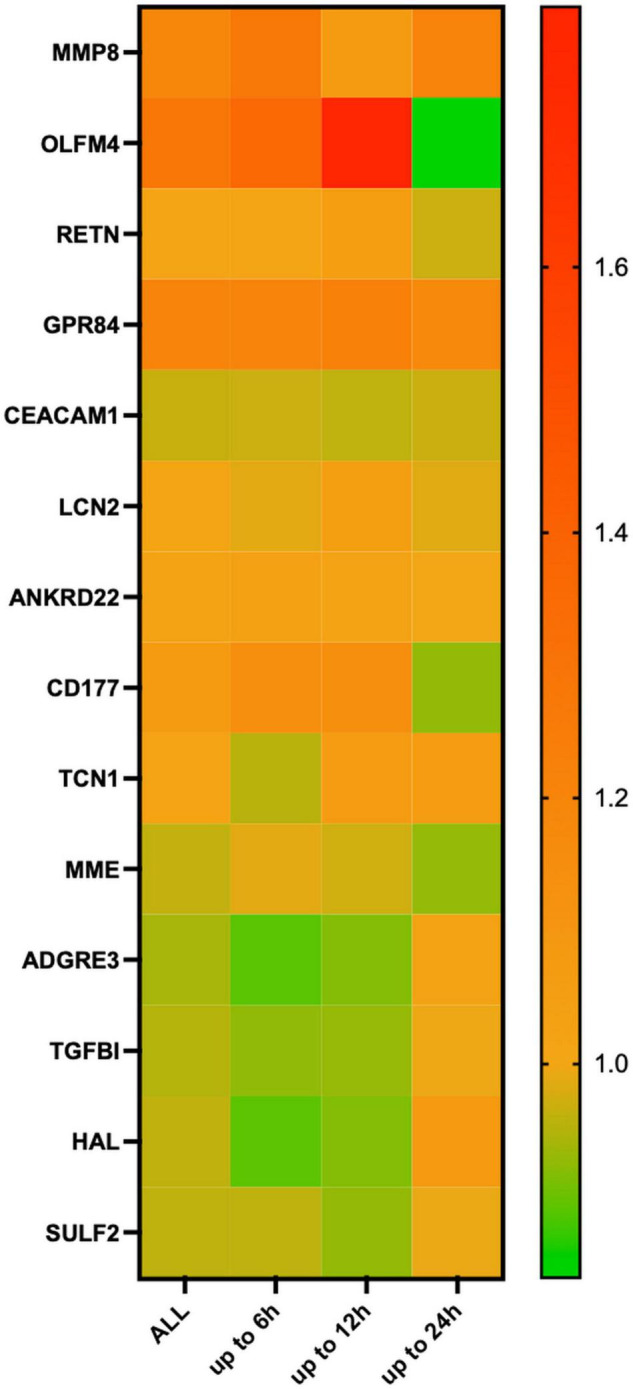
Heatmap of relative changes in means in log2 normalized count in mRNA markers, according to time point of tissue harvesting. Figure legend: 8 animals available for analysis at 6 h (Ph1 *n* = 5, Ph2 *n* = 3), 7 at 12 h (Ph1 *n* = 4, Ph2 *n* = 3) and 8 animals at 24 h (Ph1 *n* = 5, Ph2 *n* = 3). Relative changes shown on the right *y*-axis were calculated as log2-normalized count of OA-IV-LPS divided by log2-normalized count of OA for each time point. Abbreviations: OA, oleic acid; OA-IV-LPS, oleic acid and lipopolysaccharide intravenously; MMP8, matrix metalloproteinase 8; OLFM4, olfactomedin; RETN, resistin; GPR84G, protein-coupled receptor 84; CEACAM1, CEA cell adhesion molecule 1; LCN2, lipocalin 2; ZDHHC18, zinc finger dhhc-type palmitoyltransferase 18; ANKRD22, ankyrin repeat domain 22; CD177, CD177 molecule; TCN1, transcobalamin 1; MME, membrane metalloendopeptidase; RBP7, retinol binding protein 7; ADGRE3, adhesion G protein-coupled receptor E3; TGFBI, transforming growth factor beta induced; HAL, histidine ammonia-lyase; and SULF2, sulfatasse 2.

Differentiation according to observation time revealed that MMP8, GPR84, and ANKRD22 expression was higher at all time points among Ph2 and MME, TGFBI, and SULF2 among Ph1 animals. Furthermore, OLFM4 and CD177 expression was higher in Ph2 at all time points except at 24 h, and the same was shown for ADGRE3 and HAL in Ph1. At 6 h, LCN2, RETN, and TCN1 showed lower expression levels in Ph2 than Ph1 (Figure 3 and Supplementary Figure 3).

## Discussion

In two different ovine ARDS phenotypes, mimicking some key features of the presumably inflammatory subgroups in human ARDS (10), we aimed to validate the most differentially expressed mRNA markers in human ARDS subphenotypes (12).

We report two major findings: *First*, 13 of the 14 genetic markers tested in ovine lung tissue have shown similar trends in ovine Ph2/Ph1 phenotypes as reported in human blood leukocytes in the MARS cohort (12). However, only 3 markers reached statistical significance, most likely due to small samples sizes per assessed group. *Second*, mRNA expression among tissue harvesting time points showed some differences, indicating that the gene expression in ARDS phenotypes is time-dependent over the course of the disease.

Our results indicate that similar pathways might be involved (e.g., oxidative phosphorylation, NF-κB pathway) in human and ovine ARDS subgroups. This assumption has been underlined in a recent analysis, showing important similarities in gene expression patterns in human tracheal aspirate in P2 with animal models of ARDS induced by LPS (11). In the MARS cohort, mRNA markers of oxidative phosphorylation (MMP8) and neutrophil activation and function (MMP8, OLFM4, LCN2, and CD177) were shown to be a hallmark of the hyperinflammatory (or “reactive”) subphenotype (12). This finding adds evidence to the hypothesis that differentiation among presumably inflammatory ARDS subphenotypes is likely based on inflammatory pathways.

NF-κB is an important pathway in clinical ARDS (16) and LPS is known to cause NF-kB induction (17). Therefore, the higher expression in Ph2 animals of OLFM4, LCN2, TCN1, ANKRD22, RETN, and ANKRD22 can at least partially be explained by the injury model caused by OA and additional LPS IV. It also needs to be considered that because of the additional LPS injury, our ovine Ph2 model might reflect more a sepsis-related ARDS model, potentially better comparable to the MARS cohort than other described ARDS populations but not to other ARDS cohorts.

Deviation from matching trends in ovine mRNA levels as compared to human ARDS subphenotypes was mostly seen in animals assessed at 24 h. Potential explanations for this finding are that mRNA expression is likely to change over time in the dynamic biological process of ARDS. However, this assumption could not be assessed in the MARS cohort with early sampling within 24 h of ICU admission. As the animals assessed at 24 h were all part of the second study, completed 2 years after the first one, the time gap between the completion of the two studies has to be considered: the same breed of sheep but a different flock was used for experiments in study 2, this could explain parts of the reported heterogeneity. Additionally, while the induction of ARDS phenotypes and the intra-experimental handling was the same, a potential influence of sample age between between study 1 and 2 on the expression results at 24 h cannot be excluded.

Some important limitations of our study have to be considered: *First*, mRNA expression has previously been reported in peripheral leukocytes (12) rather than lung tissue. We can safely assume that mRNA expression in circulation differs from the alveolar space but to what extent cannot be determined in this study as we did not have mRNA in plasma available for analysis. *Second*, the number of biological replicates per time point is limited and consequently so is the translatability of results. *Third*, as the observed changes in gene expression may not reflect translation into proteins, any conclusion about underlying biological mechanisms is limited. *Fourth*, for a more homogenous view, we combined lung tissue from upper, middle and lower lobes of the right lung. However, due to the heterogeneous affection of different parts of the lungs in ARDS, localized differences in mRNA expression should also be investigated. *Fifth*, we did not sample for blood cultures, therefore we cannot exclude the development of an infection over the course of the study, potentially introducing a bias in the later results. *Sixth*, since all studied animals were female, we cannot determine whether the factor gender might contribute to the findings.

## Conclusion

Thirteen of 14 mRNA markers, overexpressed in human P2 and P1 ARDS subphenotypes, respectively, revealed similar expression patterns in our ovine model of ARDS phenotypes.

## Data Availability Statement

The datasets presented in this study are publicly available in the GEO repository, accession number: GSE206888.

## Ethics Statement

The animal study was reviewed and approved by Queensland University of Technology, Brisbane, Australia: Office of Research Ethics and Integrity.

## Author Contributions

KW, KH, JM, JF, and JS: conception and design of work, analysis and interpretation of data. KW, KH, JM, SL, MP, MB, EW, GL, JF, and JS: acquisition of data, drafting the work or revising it critically for important intellectual content, final approval of the version submitted for publication, and agreement to be accountable for all aspects of the submitted work. All authors contributed to the article and approved the submitted version.

## Conflict of Interest

KW has received research funding from the Julia und Gottfried Bangerter-Rhyner Stiftung, the Prince Charles Hospital Foundation and the Wesley Medical Research Foundation. In addition, she received a Ph.D. scholarship from the University of Queensland. Samantha Livingstone received a Ph.D. scholarship of the Prince Charles Foundation. GL has received research funds, through his affiliated institution from Fisher & Paykel. JS has received an Advance Queensland Industry Research Fellowship and JF received grant funding from Fisher & Paykel, the Prince Charles Hospital Foundation and the Wesley Medical Research Foundation. KW, KH, JM, SL, MP, MB, EW, GL, JF, and JS are members of the Critical Care Research Group.

## Publisher’s Note

All claims expressed in this article are solely those of the authors and do not necessarily represent those of their affiliated organizations, or those of the publisher, the editors and the reviewers. Any product that may be evaluated in this article, or claim that may be made by its manufacturer, is not guaranteed or endorsed by the publisher.

## Abbreviations

ADGRE3: adhesion G protein-coupled receptor E3
ANKRD22: ankyrin repeat domain 22
ARDS: acute respiratory distress syndrome
CD177: cluster of differentiation 177 molecule
CEACAM1: CEA cell adhesion molecule 1
GPR84: G protein-coupled receptor 84
HAL: histidine ammonia-lyase
LCN2: lipocalin 2
LIS: lung injury score
LPS: lipopolysaccharide
MARS: molecular diagnosis and risk stratification of sepsis
MME: membrane metalloendopeptidase
MMP8: matrix metalloproteinase 8
mRNA: messenger ribonucleic acid
NFkB: nuclear factor kappa-light-chain-enhancer of activated B cells
OA: oleic acid
OA-IV-LPS: oleic acid and lipopolysaccharid intravenously
OLFM4: olfactomedin 4
P1: human hypoinflammatory subphenotype
P2: human hyperinflammatory subphenotype
Ph1: ovine hypoinflammatory phenotype
Ph2: ovine hyperinflammatory phenotype
PF: PaO_2_/FiO_2_ ratio
RBP7: retinol binding protein 7
RETN: resistin
SD: standard deviation
SULF2: sulfatase 2
TCN1: transcobalamin 1
TGFBI: transforming growth factor beta induced
ZDHHC18: zinc finger DHHC-type palmitoyltransferase 18.

## References

[1] Ashbaugh DG Bigelow DB Petty TL Levine BE. Acute respiratory distress in adults. *Lancet.* (1967) 2:319–23. 10.1016/S0140-6736(67)90168-7 4143721

[2] Bellani G Laffey JG Pham T Fan E Brochard L Esteban A Epidemiology, patterns of care, and mortality for patients with acute respiratory distress syndrome in intensive care units in 50 countries. *J Am Med Assoc.* (2016) 315:788–800. 10.1001/jama.2016.0291 26903337

[3] Santacruz CA Pereira AJ Celis E Vincent J. Which multicenter randomized controlled trials in critical care medicine have shown reduced mortality? A systematic review. *Crit Care Med.* (2019) 47:1680–91. 10.1097/CCM.0000000000004000 31567349

[4] Calfee CS Delucchi K Parsons PE Taylor B Ware LB Matthay MA. Latent class analysis of ARDS subphenotypes: analysis of data from two randomized controlled trials. *Lancet Respir Med.* (2014) 2:611–20. 10.1016/S2213-2600(14)70097-9 24853585 PMC4154544

[5] Famous KR Delucchi K Ware LB Kangelaris KN Liu KD Thompson BT Acute respiratory distress syndrome subphenotypes respond differently to randomized fluid management strategy. *Am J Respir Crit Care Med.* (2017) 195:331–8. 10.1164/rccm.201603-0645OC 27513822 PMC5328179

[6] Bos LD Schouten LR van Vught LA Wiewel MA Ong DSY Cremer O Identification and validation of distinct biological phenotypes in patients with acute respiratory distress syndrome by cluster analysis. *Thorax.* (2017) 72:876–83. 10.1136/thoraxjnl-2016-209719 28450529 PMC5964254

[7] Sinha P Delucchi KL Thompson BT McAuley DF Matthay MA Calfee CS. Latent class analysis of ARDS subphenotypes: a secondary analysis of the statins for acutely injured lungs from sepsis (SAILS) study. *Intensive Care Med.* (2018) 44:1859–69. 10.1007/s00134-018-5378-3 30291376 PMC6317524

[8] Calfee CS Delucchi KL Sinha P Matthay MA Hackett J Shankar-Hari M Acute respiratory distress syndrome subphenotypes and differential response to simvastatin: secondary analysis of a randomised controlled trial. *Lancet Resp Med.* (2018) 0:1–8. 30078618 10.1016/S2213-2600(18)30177-2 PMC6201750

[9] Yehya N. Lessons learned in acute respiratory distress syndrome from the animal laboratory. *Ann Transl Med.* (2019) 7:503–503. 10.21037/atm.2019.09.33 31728356 PMC6828793

[10] Millar JE Wildi K Bartnikowski N Bouquet M Hyslop K Passmore MR Characterizing preclinical sub-phenotypic models of acute respiratory distress syndrome: an experimental ovine study. *Physiol Rep.* (2021) 9:e15048. 10.14814/phy2.15048 34617676 PMC8495778

[11] Sarma A Christenson SA Zha BS Oliveira A Neyton LPA Mick E Hyperinflammatory ARDS is characterized by interferon-stimulated gene expression, T-cell activation, and an altered metatranscriptome in tracheal aspirates. *medRxiv.* (2022). [Preprint]. 10.1101/2022.03.31.22272425

[12] Bos LD Scicluna BP Ong DY Cremer O van der Poll T Schultz MJ. Understanding heterogeneity in biological phenotypes of ARDS by leukocyte expression profiles. *Am J Respir Crit Care Med.* (2019) 200:42–50. 10.1164/rccm.201809-1808OC 30645145

[13] Mercat A Richard J-CM Vielle B Jaber S Osman D Diehl J-L Positive end-expiratory pressure setting in adults with acute lung injury and acute respiratory distress syndrome: a randomized controlled trial. *J Am Med Assoc.* (2008) 299:646–55. 10.1001/jama.299.6.646 18270353

[14] Millar JE Bartnikowski N Passmore MR Obonyo NG Malfertheiner MV von Bahr V Combined mesenchymal stromal cell therapy and extracorporeal membrane oxygenation in acute respiratory distress syndrome. A randomized controlled trial in sheep. *Am J Respir Crit Care Med.* (2020) 202:383–92. 10.1164/rccm.201911-2143OC 32293914 PMC7397785

[15] Matute-Bello G Downey G Moore BB Groshong SD Matthay MA Slutsky AS An official American thoracic society workshop report: features and measurements of experimental acute lung injury in animals. *Am J Resp Cell Mol Biol.* (2011) 44:725–38. 10.1165/rcmb.2009-0210ST 21531958 PMC7328339

[16] Moine P McIntyre R Schwartz MD Kaneko D Shenkar R Tulzo Y NF-κB regulatory mechanisms in alveolar macrophages from patients with acute respiratory distress syndrome. *Shock.* (2000) 13:85–91. 10.1097/00024382-200013020-00001 10670837

[17] Chen H Bai C Wang X. The value of the lipopolysaccharide-induced acute lung injury model in respiratory medicine. *Expert Rev Respir Med.* (2010) 4:773–83. 10.1586/ers.10.71 21128752

