# Supplementary material for: Validation of Messenger Ribonucleic Acid Markers Differentiating Among Human Acute Respiratory Distress Syndrome Subgroups in an Ovine Model of Acute Respiratory Distress Syndrome Phenotypes
Source: Front Med (Lausanne). 2022 Jul 5;9:961336. doi: 10.3389/fmed.2022.961336 (PMC9295897; doi:10.3389/fmed.2022.961336)
Supplement: Supplementary file 1 [file Data_Sheet_1.docx]

**SUPPLEMENTAL MATERIAL**

**SUPPLEMENTAL METHODS**

1. **Short study description: a) CELTIC and b) STARDUST**
2. **Quality and concentration of mRNA after extraction: eTable 1**
3. **Nanostring probe sequences: eTable 2**

**SUPPLEMENTAL RESULTS**

1. **Baseline data all animals (before induction of lung injury): eTable 3**
2. **Clinical and laboratory parameters at 6, 12 and 24 hours: eTable 4**
3. **Cytokine levels in plasma among Ph1 and Ph2 at different time points: eFigure 1**
4. **Lung Injury Score (LIS) among Ph1 and Ph2: eFigure 2**
5. **Levels of mRNA markers among different time points of tissue harvesting: eFigure 2**

**SUPPLEMENTAL REFERENCES**

1. **Study aims and methods: CELTIC and STARDUST**

The CELTIC (Combining Extracorporeal Life Support and Cell Therapy In Critical Illness) study was a preclinical ovine study to determine the safety and efficacy of mesenchymal stromal cell therapy in ovine ARDS supported by extracorporeal membrane oxygenation (ECMO). The study was performed between 01/2017 until 07/2018 (ethics approval number QUT1600001108) at the Medical Engineering Research Facility in Brisbane, Australia (1). For this analysis, only control animals with ARDS but without ECMO support or stem cell treatment were assessed (n=12).

The STARDUST (The Significance of different Subphenotypes in Acute Respiratory Distress Syndrome for Effective Treatment) was a randomized blinded preclinical study to assess treatment efficacy in phenotypes of ovine ARDS (ethics approval number QUT1800000606). For this analysis, animals in the pilot study, observed up to 24 hours, were analyzed (n=11).

**2. Quality and concentration of mRNA after extraction: eTable 1**

| **Study** | **Sample ID** | **Time point** | **Condition** | **RUL RIN** | **Conc. (ng/uL)** | **260/280** | **260/230** | **RML RIN** | **Conc. (ng/uL)** | **260/280** | **260/230** | **RLL RIN** | **Conc.** | **260/280** | **260/230** | |  |
| --- | --- | --- | --- | --- | --- | --- | --- | --- | --- | --- | --- | --- | --- | --- | --- | --- | --- |
| STARDUST | 2 | 24 | P1 | 7.9 | 2196.62 | 1.96 | 2.14 | 7.5 | 1005.18 | 2.03 | 2.16 | 9.1 | 975.65 | 1.98 | | 1.87 | |
| STARDUST | 7 | 24 | P1 | 6.3 | 1675.08 | 1.92 | 1.89 | 7.5 | 943.84 | 2.03 | 2.13 | 8.5 | 1117.37 | 1.99 | | 1.91 | |
| STARDUST | 10 | 24 | P1 | 8.9 | 1491.46 | 2.03 | 2.10 | 7.5 | 1460.57 | 2.03 | 2.07 | 9.7 | 1224.16 | 2.02 | | 2.1 | |
| STARDUST | 1 | 24 | P1 | 7.1 | 1299.57 | 1.96 | 2.16 | 7.3 | 1543.58 | 1.95 | 2.15 | 8.9 | 896.74 | 1.98 | | 2.15 | |
| STARDUST | 6 | 6 | P1 | 8.3 | 965.85 | 1.97 | 2.12 | 9.4 | 863.72 | 1.99 | 1.95 | 8.9 | 751.51 | 2.00 | | 1.95 | |
| STARDUST | 14 | 24 | P1 | 7.2 | 845.29 | 2.04 | 2.09 | 8.1 | 1822.91 | 2.01 | 2.08 | 7.2 | 818.91 | 2.00 | | 2.14 | |
| STARDUST | 4 | 12 | P2 | 5.2 | 1062.26 | 1.89 | 2.26 | 8.6 | 1474.32 | 1.99 | 2.12 | 8.7 | 847.00 | 2.03 | | 2.12 | |
| STARDUST | 8 | 24 | P2 | 6.9 | 1767.86 | 2.00 | 2.03 | 8.9 | 1636.72 | 2.02 | 2.09 | 8.5 | 2033.49 | 2.00 | | 2.11 | |
| STARDUST | 13 | 12 | P2 | 8.1 | 794.56 | 2.01 | 1.85 | 8.6 | 1476.62 | 2.00 | 2.08 | 9.1 | 1461.32 | 2.03 | | 2.02 | |
| STARDUST | 9 | 24 | P2 | 8.4 | 1614.84 | 2.01 | 2.10 | 7.8 | 993.86 | 2.01 | 2.13 | 8.9 | 1408.04 | 2.03 | | 2.16 | |
| STARDUST | 11 | 24 | P2 | 7.3 | 1539.06 | 1.99 | 1.97 | 9.4 | 1104.34 | 2.02 | 2.15 | 9.1 | 952.74 | 2.04 | | 2.05 | |
| CELTIC | 1 | 6 | P1 | 7.0 | 1527.98 | 1.99 | 2.14 | 7.1 | 1118.71 | 2.01 | 2.25 | 6.7 | 1300.09 | 2.06 | | 2.14 | |
| CELTIC | 2 | 6 | P1 | 6.6 | 1949.56 | 1.98 | 2.15 | 7.2 | 1005.18 | 2.03 | 2.16 | 6.4 | 1124.01 | 2.00 | | 2.25 | |
| CELTIC | 3 | 6 | P1 | 6.0 | 784.9 | 2.00 | 2.15 | 7.2 | 1564.60 | 2.00 | 2.21 | 8.2 | 1262.79 | 1.98 | | 2.22 | |
| CELTIC | 11 | 6 | P1 | 6.6 | 1581.68 | 2.00 | 2.14 | 7.3 | 1116.56 | 2.02 | 2.12 | 7.3 | 1324.82 | 1.95 | | 2.20 | |
| CELTIC | 4 | 6 | P2 | 7.2 | 1147.64 | 2.00 | 2.13 | 6.3 | 1042.06 | 2.02 | 2.13 | 6.0 | 898.46 | 2.04 | | 2.10 | |
| CELTIC | 5 | 6 | P2 | 6.6 | 940.58 | 2.02 | 2.03 | 6.1 | 807.67 | 2.05 | 2.21 | 7.0 | 922.87 | 2.04 | | 2.10 | |
| CELTIC | 17 | 12 | P1 | 7.2 | 294.79 | 1.91 | 1.76 | 6.9 | 840.77 | 2.01 | 1.88 | 6.5 | 626.39 | 2.02 | | 2.16 | |
| CELTIC | 18 | 12 | P1 | 6.3 | 278.32 | 1.95 | 2.22 | 6.9 | 440.58 | 1.96 | 1.96 | 7.7 | 1609.48 | 2.00 | | 2.17 | |
| CELTIC | 19 | 12 | P1 | 4.8 | 355.36 | 1.84 | 2.14 | 7.3 | 1089.41 | 2.04 | 2.15 | 6.4 | 671.52 | 1.96 | | 1.81 | |
| CELTIC | 21 | 12 | P1 | 7.5 | 1188.97 | 2.03 | 2.1 | 7.5 | 856.74 | 2.04 | 2.14 | 7.0 | 912.99 | 2.03 | | 1.97 | |
| CELTIC | 33G | 6 | P2 | 8.5 | 1947.75 | 1.98 | 2.15 | 8.8 | 1923.07 | 2.00 | 2.16 | 9.0 | 729.15 | 2.03 | | 2.13 | |
| CELTIC | 34G | 12 | P2 | 6.4 | 613.42 | 2.04 | 2.01 | 8.1 | 1512.56 | 2.03 | 2.18 | 7.1 | 1600.09 | 2.00 | | 2.21 | |

Abbreviations: RUL = right upper lobe; RML = right middle lobe; RLL = right lower lobe; conc. = concentration; RIN = RNA integrity number; Ph1 = hypoinflammatory subphenotype; Ph2 = hyperinflammatory subphenotype; STARDUST = The significance of different endotypes in Adult Respiratory Distress Syndrome (ARDS) for effective treatment; CELTIC = Combining Extracorporeal Life Support and Cell Therapy In Critical Illness; 260/280 and 260/230 = in nm, absorbance ratio of mRNA

**3. NanoString probe sequences: eTable 2**

| **Gene Symbol** | **Accession ID** | **Position** | **Probe** | **Probe Sequences** |
| --- | --- | --- | --- | --- |
| MMP8 | XM_004016526.4 | 399-498 | Probe A | TCAAGGATGACACGTCACTCCAGACTTTAAAGCCTGTCTCAATAAGTGCTCCTCAAGACCTAAGCGACAGCGTGAC  CTTGTTTCA |
|  |  |  | Probe B | CGAAAGCCATGACCTCCGATCACTCGGCAAAAGAAATCTGGATGTCTGCTTCTTTGTCCGAGGTCCTGTTGAAGG |
| OLFM4 | XM_004012247.1 | 971-1070 | Probe A | GTTGTTCTTATAAACTACTGTGCCACTGCCTTGGCCATAGGTAATTTGATCATCCTCTTCTTTTCTTGGTGTTGAGAA  GATGCTC |
|  |  |  | Probe B | CGAAAGCCATGACCTCCGATCACTCGTCAGGTTAATTTTGGCCATATTCCGGGTGTTGTACCAATTGACATACAT |
| RETN | NM_001306111.1 | 124-223 | Probe A | CCCCGGGAGGTGACACTCTGGCAGTCCAGGCCAATGCACAATTCTGCGGGTTAGCAGGAAGGTTAGGGAAC |
|  |  |  | Probe B | CGAAAGCCATGACCTCCGATCACTCCACACGTGCAGCTAGTGACGGCGAAGCCTGAAGGGCAGGTGACCAGGGAC |
| GPR84 | XM_004006274.2 | 2452-2551 | Probe A | TGATGTCAGAAGCGTTCCACATGATGGAAGCTGAGTCTCCCAGCCTTCAACTGTTGAGATTATTGAGCTTCATCATG  ACCAGAAG |
|  |  |  | Probe B | CGAAAGCCATGACCTCCGATCACTCAACTGCCACGTAACGATAGCCCAGCACAGACTCATGGTAGCAGGAGAAGC |
| CEACAM1 | XM_004015280.4 | 1104-1203 | Probe A | GAGGTCAGAGGAGCTTTCTGGTGTCGATTGATATTTCACATGCAGCCTGACAAAGACGCCTATCTTCCAGTTTGATC  GGGAAACT |
|  |  |  | Probe B | CGAAAGCCATGACCTCCGATCACTCCCACCCCAGCCACGGCGCCAATCACGATGCCGACAATGGCGCCCACTGA |
| LCN2 | XM_004005608.1 | 218-317 | Probe A | TGGAGGTGACGTTGTAGCTGCCGTCTTCCTTCAGCTCGTAGCGAACCTAACTCCTCGCTACATTCCTATTGTTTTC |
|  |  |  | Probe B | CGAAAGCCATGACCTCCGATCACTCTGGGACAAAAGTTCTGATCCAGTAGTCACAGCGCTCGTCCCTTAACAGGA |
| ZDHHC19 | XM_004003002.1 | 468-567 | Probe A | CCAGCATGGCACCCGAGTAGAGGCATAAGGACAGGACAAGCAGCATGAAGCCAATTTGGTTTTACTCCCCTCGATT  ATGCGGAGT |
|  |  |  | Probe B | CGAAAGCCATGACCTCCGATCACTCCATGGAGAAGGATAGGTGGGTCGTACGCACCAGGAAGATCAGGCAGGTGA |
| TCN1 | XM_004016508.3 | 866-965 | Probe A | CCCTGAGAAATGTTGTCAAATACTGTGCGCAGAGTTTCTCGGCAATTTCTCTTTCGGGTTATATCTATCATTTACTTG  ACACCCT |
|  |  |  | Probe B | CGAAAGCCATGACCTCCGATCACTCCCATCAAGGCAGGTAAGATCTGGGCTGCAGCAATCGGCAGGTAAAATACT |
| MME | XM_004003231.1 | 2087-2186 | Probe A | TTTTAACATAGTTTTGATAGGCTCTGTATGCTTGGCCAATACCACCATTACAACAGCCACTTTTTTTCCAAATTTTGC  AAGAGCC |
|  |  |  | Probe B | CGAAAGCCATGACCTCCGATCACTCTTGTCTGTGATTTAGGTCAAGTCCAGGAAGTAACTTTTCTTCACCATGCT |
| ADGRE3 | XM_012144956.2 | 811-910 | Probe A | TTCAAGCTGAAGGTGACTTCCTTGGAGCAGTTGTCTGTAACAACTCGAGTCACCGTGTGGACGGCAACTCAGAGAT  AACGCATAT |
|  |  |  | Probe B | CGAAAGCCATGACCTCCGATCACTCTTCCCTGGATGACATCTTTGCAATGGATTTCCATGGAGTTCACTTGGGCA |
| RBP7 | XM_004013735.4 | 213-312 | Probe A | CTTCTCCGACCTTAAATTTCACAACATAGTTCCTGAAGGTGCTGTTCGTGCCTGGAGTTTATGTATTGCCAACGAGT  TTGTCTTT |
|  |  |  | Probe B | CGAAAGCCATGACCTCCGATCACTCTTTTATCTTGCATTTTCTGTTATCCATGCCTTTGTTATCCCCATCAACTT |
| TGFBI | XM_004008814.1 | 959-1058 | Probe A | CAAATAGCGTCTTGGCTGAGTCGGGGATGAGCAGCTCGTCGATGAAGTGGCAGATAAGGTTGTTATTGTGGAGGA  TGTTACTACA |
|  |  |  | Probe B | CGAAAGCCATGACCTCCGATCACTCTTGTCGGAAAAGGTCAATGGCTGTGGAAACATCGGAGTCCGCAGCCAACT |
| HAL | XM_004007507.1 | 1334-1433 | Probe A | TTCACCATGGAAGTTTCCTCCAGAAATAGTCTCTCCCCTGCTGGCAAAGACTTCCTTCCTGTGTTCCAGCTACAAAC  TTAGAAAC |
|  |  |  | Probe B | CGAAAGCCATGACCTCCGATCACTCGCAAGTTCATGGACACCAATGGCCAAATAGTCCAGGGCTTTGGCTGGATA |
| ANKRD22 | XM_004020017.4 | 561-660 | Probe A | TGCAGCCATAACAGTCTGTGGCATTAACTTCGACACCAGCATTAAGCAGCCATTTGGAATGATGTGTACTGGGAATAAGACGACG |
|  |  |  | Probe B | CGAAAGCCATGACCTCCGATCACTCCAGAGGAATGAGAGTCTGGTTCTTCATTTCACAGGCGTAGTGTAGCGCAG |
| SULF2 | XM_004014874.1 | 1257-1356 | Probe A | GCTTGAGGATGGATTTCCCATCCATATCCGAGGGGATGTCCAGCCCACAAGAATCCCTGCTAGCTGAAGGAGGGT  CAAAC |
|  |  |  | Probe B | CGAAAGCCATGACCTCCGATCACTCCAGCTTCTTTTTCAAGTGAAACCGATTTGCCGGCCGCTCCGTGTCCAGTA |
| CD177 | XM_004015298.1 | 571-670 | Probe A | CTGCAAGTCTCCCGCAGGCTGATGGGCCCGATTTCCTGCTTGACGTAGATTGCTATCAGGTTACGATGACTGC |
|  |  |  | Probe B | CGAAAGCCATGACCTCCGATCACTCGAGACATCTGCAGTATGATCCCCCGATAACACGTCAGAACACCTTTAGGG |
| MAU2 | XM_004009141.1 | 534-633 | Probe A | GCTTCAGGCACGGCTTCACGCTCTTTACCTGCCCAGCATACGAAATTTGAGCAAGCAATTGAAGGCTTAGA |
|  |  |  | Probe B | CGAAAGCCATGACCTCCGATCACTCCATCGTGCAGCGTGGAGATGGTCTGGATGCACTGCTGCAGCT |
| POLR2A | XM_004013289.4 | 2424-2523 | Probe A | CAATGCCAATGGTATGACCCTCAATGAGGAGCCAGTTGTTGATGACAGTCCTATCAGCTAATAGGGTCGGCTCAA  CAGTGTATCC |
|  |  |  | Probe B | CGAAAGCCATGACCTCCGATCACTCAATCGTGTTCTGAATGTCTTGGTAAGTCTTAGAATCAGCGATGGAGTCCC |
| PGK1 | NM_001142516.1 | 255-354 | Probe A | GAGTTCTACAGCAACTGGCTGCAAGGAGTACTTATCAGGCATGGGGACACCTATCAATTCGTGACCCCGATCATCC  AGTCCAGAA |
|  |  |  | Probe B | CGAAAGCCATGACCTCCGATCACTCGGGCCCACACAGTCCTTCAAGAACAAAACATCCTTGCCCAGCAGAGATTT |
| RPL19 | XM_015089125.1 | 336-435 | Probe A | TTCACGGTATCGTCTGAGCAGCCGGTGCAGAATTCTCATCCTCCTCATCCTTGAGCTCTAGGCCCAAAACGACCTAATGGTCA |
|  |  |  | Probe B | CGAAAGCCATGACCTCCGATCACTCCCCTTCACCTTCAGGTACAGGCTGTGATACATGTGGCGGTCAATCTTAGA |
| TBP | XM_015097549.1 | 968-1067 | Probe A | ATCTTGAAGTCCAGGAACTTAGCTGGAAAGCCCAACTTCTGTACAACTCTCTAGCCCAGATCCTACGAGATGAGCT  ACGTAACTA |
|  |  |  | Probe B | CGAAAGCCATGACCTCCGATCACTCGGAACTTCACATCACAGCTCCCCACCATGTTCTGA |
| YWHAZ | NM_001267887.1 | 304-403 | Probe A | TAGAAGACTTTGCTCTCTGCTTGTGAAGCGTTTGGGATCAAGAACTTTTCCAAATGCACTCTATATGGAGGGAGAG  TAGCTGGAT |
|  |  |  | Probe B | CGAAAGCCATGACCTCCGATCACTCCTGCAACCTCAGCCAAGTAGCGATAGTAGTCTCCTTTCATTTTCAAA |

**4. Baseline data all animals (before induction of lung injury): eTable 3**

|  | **All**  (n=23) | **Ph1 (OA)**  (n=14) | **Ph2 (OA-IV-LPS)**  (n=9) |
| --- | --- | --- | --- |
| Weight (kg) | 49 (47-52) | 50 (48-53) | 48 (43-52) |
| **Hemodynamics** |  |  |  |
| Heart rate (bpm) | 99 (±21) | 97 (±24) | 102 (±17) |
| Mean arterial pressure (mmHg) | 115 (84-120) | 105 (83-122) | 119 (98-124) |
| Mean pulmonary arterial pressure (mmHg) | 17 (±4) | 18 (±4) | 16 (±4) |
| **Blood gas analysis** |  |  |  |
| PF ratio | 412 (301-511) | 412 (333-510) | 444 (225-521) |
| Oxyhaemoglobin (%) | 94.2 (±11.5) | 96.2 (±3.9) | 91.2 (±17.9) |
| PaCO_2_ (mmHg) | 42.8 (±8.9) | 42.2 (±6.1) | 43.6 (±12.6) |
| Bicarbonate (mEq/L) | 25.1 (±2.6) | 25.0 (±3.0) | 25.3 (±2.1) |
| Base excess (mEq/L) | 0.7 (±3.3) | 0.6 (±3.4) | 0.9 (±3.3) |
| **Full blood count** |  |  |  |
| Haemoglobin (g/L) | 98 (±19) | 97 (±18) | 99 (±20) |
| Platelets (x 10^^9^/L) | 255 (±84) | 288 (±71) | 211 (±82) |
| White cell count (x 10^^9^/L) | 5.3 (±1.9) | 5.3 (±1.4) | 5.2 (±2.6) |
| Lymphocyte count (x 10^^9^/L) | 2.8 (±1.2) | 2.7 (±1.0) | 3.1 (±1.7) |
| Neutrophil count (x 10^^9^/L) | 2.0 (±0.9) | 2.2 (±1.0) | 1.8 (±0.8) |
| **aPTT** (s) | 27 (±6) | 30 (±7) | 24 (±4) |
| **Biochemistry** |  |  |  |
| Sodium (mmol/L) | 142 (±2.4) | 142 (±2) | 143 (±2) |
| Potassium (mmol/L) | 4.2 (±0.4) | 4.1 (±0.4) | 4.4 (±0.4) |
| Albumin (g/L) | 32 (±4) | 32 (±3) | 32 (±5) |
| AST (IU/L) | 87 (±17) | 89 (±19) | 84 (±16) |
| ALP (IU/L) | 149 (±68) | 117 (±35) | 192 (±79) |
| Bilirubin (umol/L) | 2.9 (±0.9) | 2.8 (±0.8) | 2.9 (±1.1) |
| CK (IU/L) | 167 (±42) | 169 (±41) | 163 (±46) |
| Creatinine (mmol/L) | 0.07 (0.06-0.08) | 0.07 (0.07-0.08) | 0.07 (0.06-0.09) |

Parameters displayed as mean and standard deviation or median and IQR

Abbreviations: OA = Oleic Acid; OA-IV-LPS = Oleic Acid and lipopolysaccharide intravenously; Ph1 and Ph2 = phenotype 1 and 2; PF ratio = PaO_2_/FiO_2_ ratio; PaCO_2_ = arterial CO2 partial pressure ; aPTT = activated partial thromboplastin time; AST = aspartate aminotransferase; ALP = alkaline phosphatase; CK = creatinine kinase

**5. Clinical and laboratory parameters at 6, 12 and 24 hours: eTable 4**

|  | **at T6** | | **at T12** | | **at T24** | |
| --- | --- | --- | --- | --- | --- | --- |
|  | **Ph1, n=14** | **Ph2, n=9** | **Ph1, n=9** | **Ph2, n=6** | **Ph1, n=5** | **Ph2, n=3** |
| Temperature (°C) | 39.1 (1.1) | 38.7 (1.3) | 39.1 (1.0) | 39.0 (1.4) | 38.6 (0.6) | 39.1 (0.9) |
| **Hemodynamic parameters** |  |  |  |  |  |  |
| Mean arterial blood pressure (mmHg) | 75 (19) | 61 (2) | 77 (14) | 59 (6) | 73 (7) | 77 (6) |
| Heart rate (bpm) | 113 (24) | 119 (27) | 88 (19) | 96 (34) | 78 (13) | 96 (33) |
| Use of noradrenaline (mcg/kg/min) | 0.09 (0.15) | 0.30 (0.28) | 0.13 (0.12) | 0.25 (0.16) | 0.14 (0.15) | 0.17 (0.21) |
| **Mechanical ventilation** |  |  |  |  |  |  |
| Minute ventilation (L/min) | 10.6 (2.3) | 11.0 (2.2) | 10.3 (1.9) | 10.9 (2.8) | 10.0 (3.2) | 12.5 (2.9) |
| Compliance (mL/cmH_2_O) | 18.6 (6.1) | 19.9 (4.7) | 23.5 (6.3) | 16.4 (1.8) | 25.4 (12.4) | 18.2 (3.6) |
| Plateau pressure (cm H_2_O) | 28 (7) | 25 (3) | 24.4 (4.2) | 24.6 (3.4) | 20.8 (4.3) | 22.1 (3.5) |
| **Blood gases** |  |  |  |  |  |  |
| PaO2/FiO2 ratio | 172 (155) | 250 (123) | 198 (128) | 238 (118) | 272 (112) | 254 (80) |
| PaCO2 (mmHg) | 58 (26) | 49 (4) | 44 (9) | 63 (22) | 16 (9) | 49 (9) |
| Lactate | 3.5 (4.8) | 5.5 (3.9) | 1.2 (1.0) | 5.7 (2.6) | 1.3 (0.5) | 3.2 (1.5) |
| Base excess (mmol/L) | -2.3 (6.8) | -2.0 (3.7) | 0.6 (5.2) | -4.3 (4.3) | 1.1 (1.9) | -3.1 (4.2) |
| **Full blood count and biochemistry** |  |  |  |  |  |  |
| Hemoglobin (g/L) | 101 (18) | 120 (14) | 98 (16) | 112 (17) | 100 (20) | 111 (2) |
| Platelets (10^9/L) | 246 (51) | 184 (107) | 245 (58) | 101 (50) | 250 (50) | 97 (89) |
| Neutrophil count (10^9/L) | 1.7 (0.9) | 0.4 (0.2) | 1.7 (0.8) | 2.5 (3.6) | 1.0 (0.6) | 3.36 (4.9) |
| Creatinine (mmol/L) | 0.08 (0.02) | 0.08 (0.02) | 0.08 (0.01) | 0.12 (0.04) | 0.1 (0.008) | 0.14 (0.05) |
| Bilirubin (umol/L) | 2 (1) | 5 (4) | 10 (9) | 11 (6) | 28 (9) | 26 (4) |

All animals alive at this time point were assessed. Parameters displayed as median and IQR, cytokine levels as mean and standard deviation

Abbreviations: Ph1 and Ph2 = phenotype 1 and 2; PaCO2 (mmHg) = arterial carbondioxide partial pressure

**6. Cytokine levels in plasma among Ph1 and Ph2 at different time points: eFigure 1**


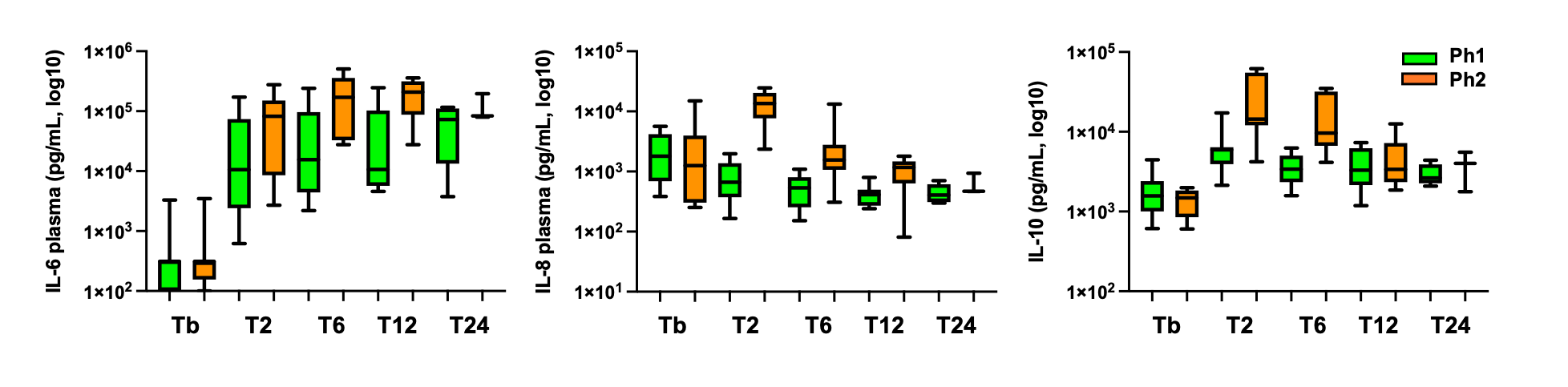


Abbreviations: Ph1 and Ph2 = phenotype 1 and 2; T = time point; IL = interleukin

**7. Lung Injury Score (LIS) among Ph1 and Ph2: eFigure 2**

**
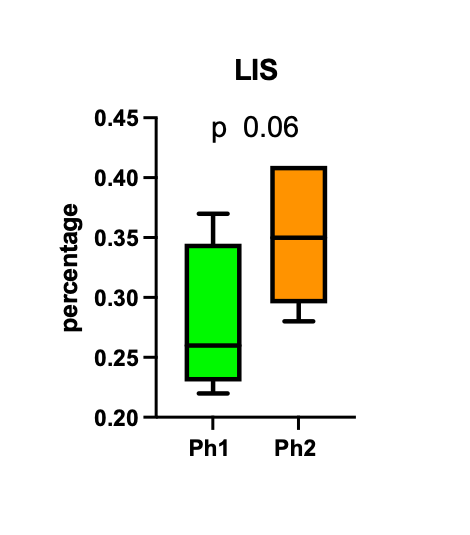
**

Abbreviations: Ph1 and Ph2 = phenotype 1 and 2; LIS = Lung Injury Score

**8. Levels of mRNA markers among different time points of tissue harvesting: eFigure 3**

**
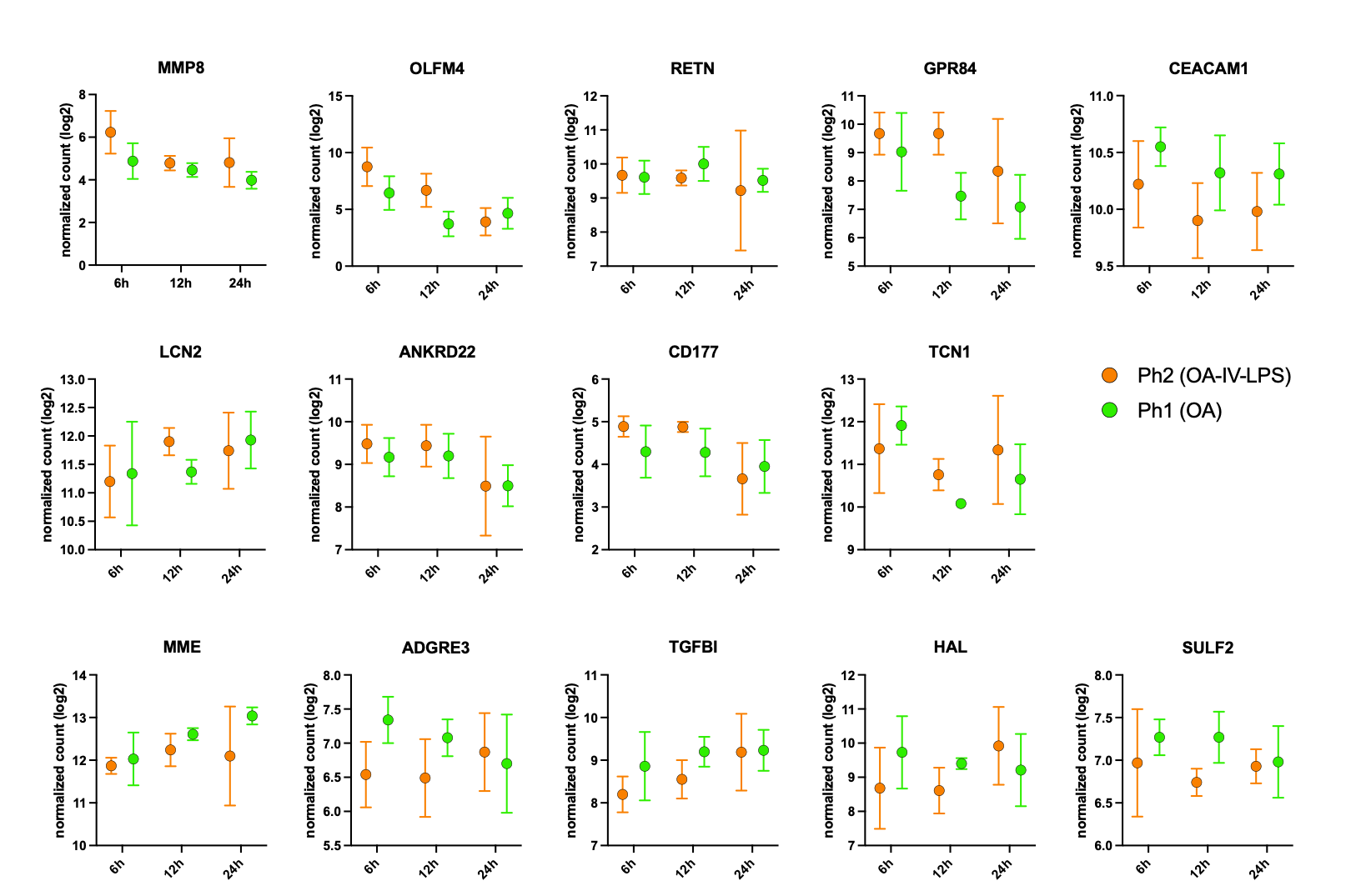
**

Error bar displays standard deviation

Abbreviations: MMP8 = Matrix Metallopeptidase 8; OLFM4 = Olfactomedin; RETN = Resistin; GPR84G = Protein-Coupled Receptor 84; CEACAM1 = CEA Cell Adhesion Molecule 1; LCN2 = Lipocalin 2; ANKRD22 = Ankyrin repeat domain 22; CD177 = CD177 molecule; TCN1 = Transcobalamin 1; MME = Membrane Metalloproteinase; ADGRE3 = Adhesion G Protein-Coupled Receptor E3; TGFBI = Transforming Growth Factor Beta Induced; HAL = Histidine Ammonia-Lyase; SULF2 = Sulfatase

**SUPPLEMENTAL REFERENCES**

1. Millar JE, Bartnikowski N, Passmore MR, Obonyo NG, Malfertheiner MV, von Bahr V, et al. Combined mesenchymal stromal cell therapy and extracorporeal membrane oxygenation in acute respiratory distress syndrome a randomized controlled trial in sheep. Am J Respir Crit Care Med. 2020;202(3):383–92.
